# Broadband achromatic optical metasurface devices

**DOI:** 10.1038/s41467-017-00166-7

**Published:** 2017-08-04

**Authors:** Shuming Wang, Pin Chieh Wu, Vin-Cent Su, Yi-Chieh Lai, Cheng Hung Chu, Jia-Wern Chen, Shen-Hung Lu, Ji Chen, Beibei Xu, Chieh-Hsiung Kuan, Tao Li, Shining Zhu, Din Ping Tsai

**Affiliations:** 1 National Laboratory of Solid State Microstructures, College of Engineering and Applied Sciences, School of Physics, Nanjing University, Nanjing, 210093 China; 2 Research Center for Applied Sciences, Academia Sinica, Taipei, 11529 Taiwan; 3 Department of Physics, National Taiwan University, Taipei, 10617 Taiwan; 4 Department of Electrical Engineering and Graduate Institute of Electronics Engineering, National Taiwan University, Taipei, 10617 Taiwan; 5 Collaborative Innovation Center of Advanced Microstructures, Nanjing, 210093 China; 6 College of Engineering, Chang Gung University, Taoyuan, 33302 Taiwan

## Abstract

Among various flat optical devices, metasurfaces have presented their great ability in efficient manipulation of light fields and have been proposed for variety of devices with specific functionalities. However, due to the high phase dispersion of their building blocks, metasurfaces significantly suffer from large chromatic aberration. Here we propose a design principle to realize achromatic metasurface devices which successfully eliminate the chromatic aberration over a continuous wavelength region from 1200 to 1680 nm for circularly-polarized incidences in a reflection scheme. For this proof-of-concept, we demonstrate broadband achromatic metalenses (with the efficiency on the order of ∼12%) which are capable of focusing light with arbitrary wavelength at the same focal plane. A broadband achromatic gradient metasurface is also implemented, which is able to deflect wide-band light by the same angle. Through this approach, various flat achromatic devices that were previously impossible can be realized, which will allow innovation in full-color detection and imaging.

## Abstract (web summary)

Metasurfaces suffer from large chromatic aberration due to the high phase dispersion of their building blocks, limiting their applications. Here, Wang et al. design achromatic metasurface devices which eliminate the chromatic aberration over a continuous region from 1200 to 1680 nm in a reflection schleme.

## Introduction

Wavelength dispersion is a key characteristic of optical materials, which always plays an important role in designing optical components and systems. In most dielectrics like glasses, the index of refraction decreases with the longer wavelength, which is called normal chromatic dispersion^1^. Utilizing such materials, refractive lenses will perform larger focal lengths and prisms will deflect at a smaller angle for longer wavelength. Such chromatic aberration significantly degrades the performance of full-color optical applications, such as in communication, detection, imaging, displaying etc. Taking the refractive lenses as an example, complete elimination of chromatic aberration at three and four wavelengths is accomplished by integrating several materials arranged into a single component to achieve the same focal length, which are termed as apochromatic and superachromatic lens^2^, respectively. Although successful, this strategy adds weight, complexity and cost to optical systems, which greatly limits their usage.

In contrast to the traditional bulk optical elements, metasurfaces^3–6^ provide a new perspective on flexibly shaping the electromagnetic field^7^ by manipulating its phase, amplitude as well as polarization at will via a compact and easy-of-fabrication system. Such promising approaches prove their great features and functionality in a variety of applications, such as nonlinear dynamics^8^, light beam shaping^9–11^, highly dimensional holographic images^12–15^, polarization control and analysis^16–18^, invisible cloak carpet^19^ etc. In addition to the compact dimension, the main distinction between metalenses (planar lenses based on metasurfaces) and traditional optical lenses is the integrated functionality that allows the achievement of specific characteristics such as multispectral^20^, dual-functional^21^ and off-axis focusing^22^. Recent works reported the dielectric metasurfaces for metalens with high numerical aperture (NA) and optical planar camera with monochromatic aberrations that are comparable with the commercial products, presenting their potential prospect in real applications^23, 24^. However, these proposed metasurface devices still exhibit large chromatic aberration, which is mainly attributed to following fundamental properties: the resonant phase dispersion of the building blocks used in metasurfaces, the intrinsic dispersion in used materials, as well as the different phase accumulation through light propagation in free space with various wavelengths. Many works have shown that optical metasurfaces working in a relatively broad wavelength bandwidth can be achieved^25–30^. However, in these reports the optical performance of resonant elements in the metasurfaces is unchanged in a broad bandwidth and this is insufficient for the suppression of chromatic aberration.

Up to now, several pioneering works have realized multi-wavelength achromatic metalenses that are capable of eliminating the chromatic aberration at several discrete wavelengths^31–37^. Very recently, there has been a successful demonstration of an achromatic metalens which is able to suppress the chromatic effect over a 60-nm bandwidth in the visible^38^. However, although continuous, the working bandwidth (about 11.4% of the central wavelength) is still not broad enough for practical purposes and there is still lack of an effective design rule for unit elements in achromatic metasurface devices. Therefore, much work remains to be done in order to accomplish a broadband achromatic flat optical component working over a continuous bandwidth because of the abrupt phase change from the resonant unit elements. In this work, we employ the integrated-resonant unit element in metasurfaces with smooth and linear phase dispersion combining with geometric phase to design broadband achromatic flat optical components. For the proof of concept, achromatic converging metalenses and beam deflector with gradient metasurfaces are demonstrated that are valid at will within a broad infrared (IR) bandwidth from wavelength of 1200 to 1680 nm. The broadband achromatic metasurface devices based on our approach greatly innovate a complete class of designs in full-color light manipulation and imaging and push them forward to the practical applications.

## Results

### Phase requirement of broadband achromatic metalens

**Fig. 1:**
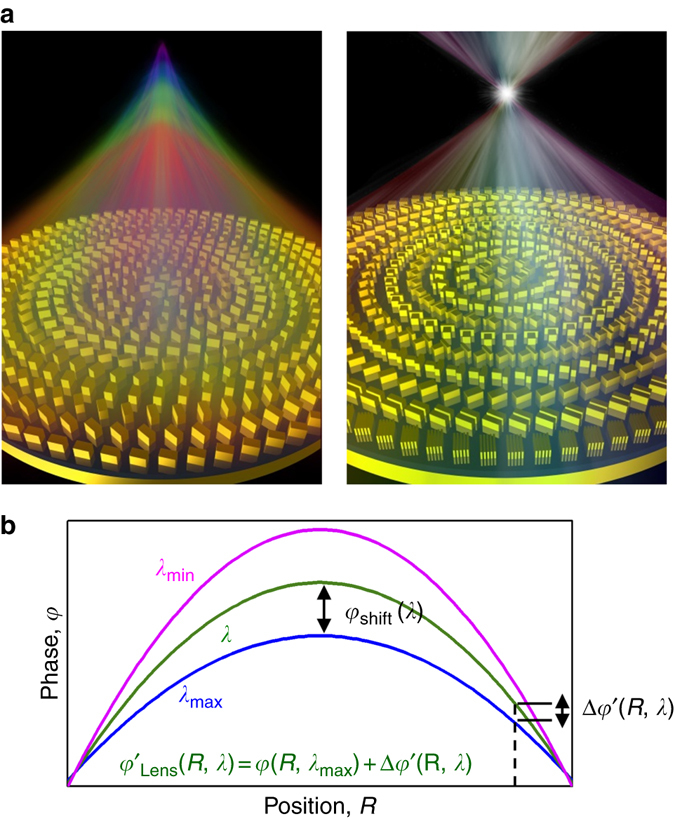
Achromatic metalens. **a** Schematic for chromatic (*left*) and achromatic (*right*) metalenses. In the case of chromatic metalens, the focal length is changed as the incident wavelength is switched, resulting in a rainbow-like focal line when it is illuminated by a light source with continuously changed wavelength. The focal point will become a single spot for the case of BAML with optimized phase compensation. **b** Phase profile for a BAML at arbitrary wavelength of *λ* ∈ {*λ*_min_, *λ*_max_}

As the most basic and important functional element, converging lens plays a crucial role in various optical systems. The typical distribution of phase retardation for a metalens follows:

1 $$\varphi \left( {R,\lambda } \right) = - \left[ {2{\rm{\pi }}\left( {\sqrt {{R^2}{\rm{ + }}{f^2}} - f} \right)} \right]\frac{{\rm{1}}}{\lambda }$$

where $R{\rm{ = }}\sqrt {x_0^2{\rm{ + }}y_0^2}$ is the distance from arbitrary position (*x*_0_, *y*_0_) on the metalens to the center and *f* is the focal length. To realize a broadband achromatic metalens (BAML), which is schematically shown in the right side of Fig. 1a, such phase retardation with a fixed focal length is exactly required in a wide range of wavelength. Generally, for the case of working wavelength ${{\lambda }} \in \left\{ {{{{\lambda }}_{{\rm{min}}}},\quad {{{\lambda }}_{{\rm{max}}}}} \right\}$, with *λ*_min_ and *λ*_max_ being the boundaries of the interested wavelength band, the phase in Eq. 1 can be re-written as:

2 $${\varphi _{{\rm{Lens}}}}\left( {R,\lambda } \right) = \varphi \left( {R,{\lambda _{{\rm{max}}}}} \right){\rm{ + \Delta }}\varphi \left( {R,\lambda } \right)$$

with

3 $$\Delta \varphi \left( {R,\lambda } \right) = - \left[ {2\pi \left( {\sqrt {{R^2} + {f^2}} - f} \right)} \right]\left( {\frac{1}{\lambda } - \frac{1}{{{\lambda _{{\rm{max}}}}}}} \right)$$

The BAML can only be realized once these two key phase characteristics are exactly satisfied simultaneously; therefore, the designed phase of BAML can be accordingly divided into two components. The former part in Eq. 2, *φ*(*R*, *λ*_max_), is considered as a basic phase profile, which is only related with *λ*_max_ and is independent to the working wavelength *λ*. Such phase profile can be acquired by exploiting the geometric phase as the phase modulation in each metalens unit element. The geometric phase, also called Berry phase, is produced from the rotation of the resonant elements in metasurface with a circularly-polarized incidence^39^. The produced phase modulation only depends on the orientation of scattered elements, which can therefore work as the solution for the wavelength-independent phase modulation. In contrary, the latter part in Eq. 2, Δ*φ*(*R*, *λ*), is a function of working wavelength and presents a linear relation with 1/*λ*, which is considered as the phase difference between various incident wavelengths. Such phase difference can be obtained by suitably designing the phase response of each unit element of the metalens, which must also exhibit the phase dispersion owning a linear relation with 1/*λ*, in consistent with Δ*φ* (*R*, *λ*). Since the mechanism of this resonant phase response is completely different from the geometric phase, these two parts of phase won’t disturb with each other, but can be simply merged together.

It should be mentioned that an additional phase shift *φ*_shift_(*λ*) can be introduced to optimize the phase compensation effect from the specially designed metalens, since such phase shift won’t affect the focusing property of the metalens^34^, as shown in Fig. 1b. Then, the phase profile of the achromatic metalens is changed to

4 $$\varphi' _{{\rm{Lens}}} \left( {R,\lambda } \right) = - \left[ {2\pi \left( {\sqrt {{R^2} + {f^2}} - f} \right)} \right]\frac{1}{\lambda } + {\varphi _{{\rm{shift}}}}(\lambda )$$

The phase difference consequently becomes ${\rm{\Delta }}\varphi '\left( {R,\lambda } \right) = {\rm{\Delta }}\varphi \left( {R,\lambda } \right){\rm{ + }}{\varphi _{{\rm{shift}}}}\left( \lambda \right)$. To maintain the linear relation with 1/*λ*, *φ*_shift_(*λ*) has to match the form as *φ*_shift_ (*λ*) = *α*/*λ* + *β*, with $\alpha = \chi \frac{{{\lambda _{{\rm{max}}}}{\lambda _{{\rm{min}}}}}}{{{\lambda _{{\rm{max}}}} - {\lambda _{{\rm{min}}}}}}$ and $\beta = - \chi \frac{{{\lambda _{{\rm{min}}}}}}{{{\lambda _{{\rm{max}}}} - {\lambda _{{\rm{min}}}}}}$, where *χ* denotes the largest additional phase shift between *λ*_min_ and *λ*_max_ at the central position of the BAML. It is the key parameter in the design of BAML, especially for the determination of the diameter of BAML. How to design special unit elements to compensate the phase difference at corresponding position is subsequently the crucial issue. Here, for a proof-of-concept demonstration, the working wavelength is chosen in the commonly used telecom region, i.e., {*λ*_min_, *λ*_max_}→{1200, 1680 nm}.

### Design of integrated-resonant unit elements

**Fig. 2:**
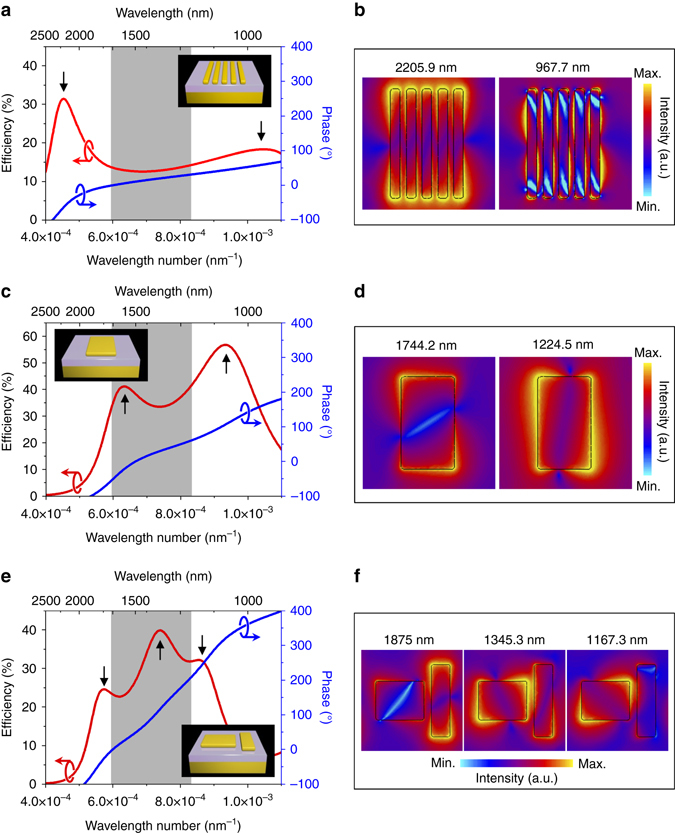
Phase profile of integrated-resonant unit elements based on coupled nano-rods. **a**, **c**, **e** RCP-to-LCP polarization conversion efficiency (*red curves*), phase profile (*blue curves*) and (**b**, **d**, **f**) electric field distribution (color mapping) of integrated-resonant unit elements. Each unit element with 550 nm periods along *x* and *y* axes consists of one or several gold MNRs with fixed thickness 30 nm. The MNRs stand on a SiO_2_/Au substrate with varying gap size, length and width are optimized to achieve various phase compensation between *λ*_min_ and *λ*_max_ (Supplementary Tables 1 and 2). The thickness of dielectric spacer SiO_2_ is 60 nm. *Gray* region and black arrows remark the range of working bandwidth of BAML and positions of resonant modes, respectively

In our design, the basic building blocks of the BAML are metallic nano-rods (MNRs) and their assemblies, coupled MNRs. The metal-dielectric-metal structural configuration (insets in Fig. 2) is implemented to access a cavity-like resonance so as to enhance the operating efficiency of metasurfaces^40, 41^. Generally, a single MNR is able to support multiple plasmonic resonances, such as the fundamental dipolar mode and various high order modes. An abrupt phase shift usually occurs when these plasmonic resonances are excited, which does not fit the requirement of smooth and linear phase difference Δ*φ*′(*R*, *λ*) (Supplementary Fig. 1). In contrast, the phase distribution between these resonances exhibits a smooth trend with a nearly linear profile against 1/*λ*. Since such state is fall in between the two strong resonant modes, it could be termed as an integrated-resonant state. In addition, such integrated-resonant features provide a promising method to slickly compensate the phase difference for BAML. Moreover, since the produced phase compensation owns a linear relation with of 1/*λ*, the BAML will naturally be achieved at any wavelength within the working bandwidth as long as the largest phase difference Δ*φ*′(*R*, *λ*_min_) is exactly compensated by the designed unit elements.

Due to linear relation of the phase distribution, the slope *S* in the phase spectrum can be directly obtained as $S = C{\rm{/}}\left( {\frac{1}{{{{\it{\lambda }}_{R{\rm{1}}}}}}} \right) - \left( {\frac{1}{{{{\it{\lambda }}_{R{\rm{2}}}}}}} \right)$, where *λ*_*R*1_ and *λ*_*R*2_ are the resonant wavelengths of the MNR, and *C* is a constant. This indicates that the slope of the phase profile can be tuned via tuning the wavelength distance between two resonances. Therefore, one can effectively steer the phase compensation *δ*(*λ*) provided by the integrated-resonant unit elements composed of MNRs or coupled MNRs, which obeys $\delta \left( \lambda \right) = S\left( {\frac{1}{\lambda } - \frac{1}{{{\lambda _{{\rm{max}}}}}}} \right) = C\left( {\frac{1}{\lambda } - \frac{1}{{{\lambda _{{\rm{max}}}}}}} \right){\rm{/}}\left( {\frac{1}{{{\lambda _{R1}}}} - \frac{1}{{{\lambda _{R2}}}}} \right)$. The largest phase compensation produced by these unit elements between *λ*_min_ and *λ*_max_ therefore becomes $\delta = C\left( {\frac{1}{{{\lambda _{{\rm{min}}}}}} - \frac{1}{{{\lambda _{{\rm{max}}}}}}} \right){\rm{/}}\left( {\frac{1}{{{\lambda _{R1}}}} - \frac{1}{{{\lambda _{R2}}}}} \right)$, which can be directly utilized to cover the largest phase difference Δ*φ*′(*R*, *λ*_min_) required at any position in BAML.

For the case of slight slope *S* corresponding to the small phase compensation, a single MNR with large aspect ratio is utilized, in which the fundamental dipole mode and high order mode are excited. When the wavelengths of these two plasmonic modes are tuned far away from each other, the small phase compensation is realized. The detail information of the single MNR structure with large aspect ratio can be found in Supplementary Fig. 2. To improve the operating efficiency, multiple MNRs are integrated within an integrated-resonant unit element. Following this approach, the smallest phase compensation, also a key parameter for determining the size of BAML, can be achieved at around 30˚, as shown in Fig. 2a. The electric field distributions at the wavelengths corresponding to the dipole mode and second order plasmonic mode are respectively plotted in Fig. 2b.

The larger phase compensation can be acquired by decreasing the difference between two resonant wavelengths of the MNR. With the aspect ratio of the MNR decreasing, the dipole mode along its short axis can be excited instead of the second order plasmonic modes, being the nearest resonant mode from the dipole mode along the long axis. With the wavelength distance between these two modes shrinking, larger phase compensation is obtained. As shown in Fig. 2c, two dipole modes can be evidently observed in the operation efficiency spectrum, in which 120˚ phase compensation is realized. The electric field distributions at these two resonant wavelengths are shown in Fig. 2d, which obviously indicates their identities.

It will turn into another issue when the phase compensation is larger than 150˚ in such integrated-resonant MNRs, in that case steering the relation between two resonances is insufficient, and one more resonance is necessary to be introduced into the working wavelength region. However, it is quite challenging to exhibit three resonances within the concerning wavelength band using single MNR. Therefore, a compound structure composed of two perpendicularly aligned MNRs with different lengths and widths is utilized to introduce the third plasmonic resonant mode. Assuming that the second resonant wavelength *λ*_*R*2_ located near the middle of resonant wavelength region {*λ*_*R*1_, *λ*_*R*3_}, the slopes of phase spectrum of two wavelength regions {*λ*_*R*1_, *λ*_*R*2_} and {*λ*_*R*2_, *λ*_*R*3_} can be close to ${S_{21}}{\rm{ = }}{C_{21}}{\rm{/}}\left( {\frac{1}{{{\lambda _{R1}}}} - \frac{1}{{{\lambda _{R2}}}}} \right)\sim{S_{32}}{\rm{ = }}{C_{32}}{\rm{/}}\left( {\frac{1}{{{\lambda _{R2}}}} - \frac{1}{{{\lambda _{R3}}}}} \right)$, assuming *C*_21_ ∼ *C*_32_ = *C*. Thus, the entire phase slope *S* can be expressed as $S \sim  2C{\rm{/}}\left( {\frac{1}{{{\lambda _{R1}}}} - \frac{1}{{{\lambda _{R3}}}}} \right)$, which can theoretically be two times larger than the single MNR case. Figure 2e presents the case of 210˚ phase compensation between *λ*_min_ and *λ*_max_ after structural optimization with three resonances. The corresponding electric field distributions are also provided to recognize which individual MNR these resonances belonging to, as shown in Fig. 2f. Based on this approach, the largest phase compensation can reach to even 360°, with a fairly good efficiency at most wavelengths (Supplementary Fig. 3). In fact, the phase compensation can be further increased by introducing more resonances to the integrated-resonant unit element, which will enable a BAML with larger size (Supplementary Fig. 10).

### Characterizations of the BAML

**Fig. 3:**
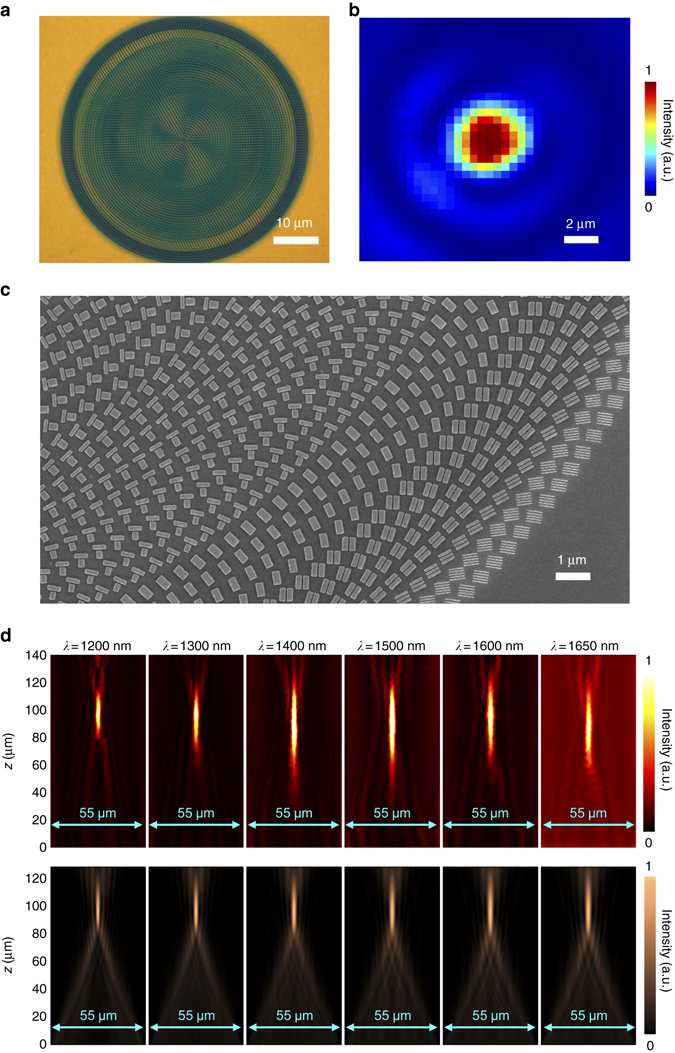
Verification of achromatic converging metalens. **a** Optical image of fabricated metalens with NA = 0.268. **b** Measured light intensity of focal spot at incident wavelength *λ* = 1500 nm. **c** Zoom-in scanning electron microscope (SEM) image of fabricated metalens. More images and design details can be found in the Supplementary Note 3. **d** Experimental (*top row*) and numerical (*bottom row*) intensity profiles of BAML along axial planes at various incident wavelengths

Employing the proposed policy, a broadband achromatic converging metalens with numerical aperture (NA) = 0.268 is designed and demonstrated. Figure 3a shows the optical image of the fabricated metalens, which has a diameter of 55.55 μm and designed focal length of 100 μm. This metalens was fabricated using standard electron beam lithography (see Methods section for details). The measured focal spots show the highly symmetric profiles, performing a good ability on light convergence in designed BAML. The focal spot at the middle of the working wavelength range, 1500 nm, is presented as an example in Fig. 3b. Figure 3c presents the zoom-in scanning electron microscope (SEM) image, in which a set of integrated-resonant MNRs with accurate gap sizes are observed. To capture the light intensity distribution, a couple of optical components including an IR objective and an IR coupled charge device (CCD) are moved together along the propagation direction of reflected light at different *x–y* planes (see Supplementary Fig. 7 for details of measurement setup). The right-hand circular polarization (RCP) to left-hand circular polarization (LCP) light intensity profiles on *x*–*z* plane is subsequently obtained from stitching all captured images together. Figure 3d shows that the measured and simulated light intensity profiles along the cross section plane are in a good agreement with each other. As theoretical predication, the focal length keeps almost unchanged while incident wavelength varies, indicating the realization of a broadband achromatic converging property in near-IR region.

**Fig. 4:**
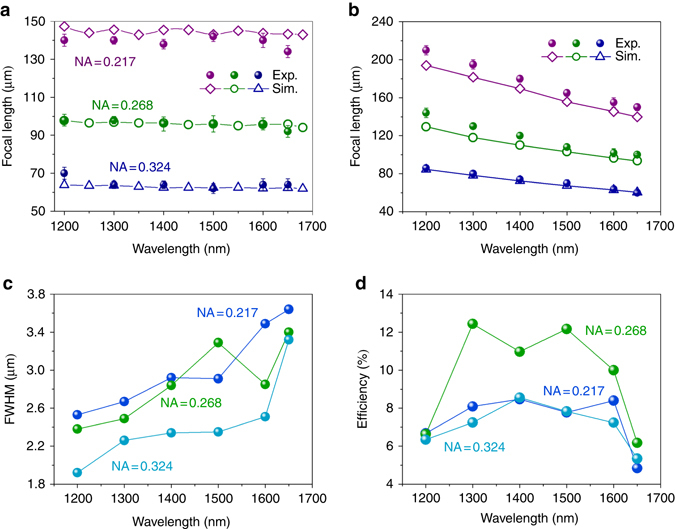
Performance of broadband achromatic metalens. Measured and simulated focal length of **a** BAML with various NA values and **b** chromatic metalenses. Measured **c** FWHM and **d** operation efficiency at corresponding focal plane as a function of wavelength. The efficiency is defined as the ratio of light intensity from the focal spot at corresponding focal plane to the light intensity reflected by a metallic mirror with the same pixel sizes. The *error bars* present the length of distance from the focal plane where the intensity of focal spot remains the maximum value in measurements

For optical characteristics of the designed metalens, the focal lengths vs. incident wavelength of both achromatic and chromatic metalenses are measured. Figure 4a shows the results of three achromatic metalenses with different NA values, which exhibits unchanged focal length with varied incident wavelength. These results confirmed again that the designed BAML is able to focus the incident light at the same focal plane in a continuous and wide range of wavelength (~33.3% of the central wavelength). Such perfect achromatic property can be realized from metalens with various NA values. To the best of our knowledge, this is the state-of-the-art demonstration of an achromatic metalens, which is able to completely eliminate the chromatic effect over the broadest wavelength range at continuously changed steps. In contrast to the BAML, the focal length of chromatic metalenses (which is designed and fabricated using Berry phase based metasurfaces) strongly depends on the incident wavelength. For comparison, the phase distributions of chromatic metalenses are designed for similar NA values with the achromatic ones at the longest wavelength in the interested wavelength range (Supplementary Note 4). The obvious chromatic aberration can be observed with evident focal length enlarging as the wavelength decreasing in all three metalenses, shown in Fig. 4b.

To further examine the working performance of designed BAML, full-width at half-maximum (FWHM) and operating efficiency are measured at near-IR region, as shown in Figs. 4c and 4d. The FWHM, which is defined as the beam waist of the normalized light intensity at 0.5, is estimated by fitting the measured light intensity along the focal line with Gaussian function. All measured FWHM are located at the range of 1.5*λ*~2*λ* that are nearly diffraction-limited. The maximum focusing efficiency of 8.4, 12.44 and 8.56% were measured for BAML with NA = 0.217, 0.268 and 0.324, respectively. The focusing efficiency drops at the boundaries of measured wavelength region is due to the lower polarization conversion efficiency of utilized unit elements at *λ*_min_ and *λ*_max_. Though the efficiency spectrum shows some fluctuations in the operating wavelength range, which is the inevitable corollary from the introduction of the integrated-resonant unit elements, the average working efficiency can be further increased by replacing the material components with dielectrics or further optimizing the structural configuration^42, 43^.

### Design and characterizations of the broadband achromatic gradient metasurface

**Fig. 5:**
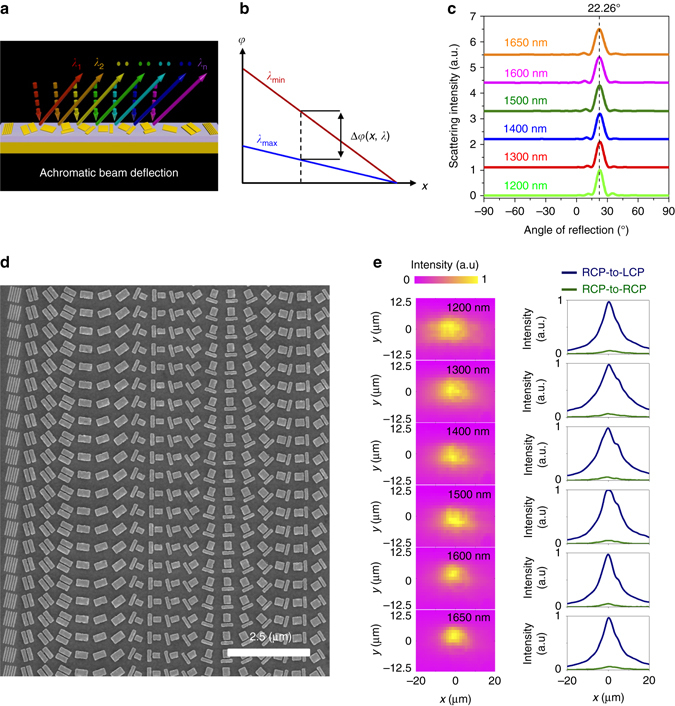
Broadband achromatic gradient metasurfaces. **a** Schematic of beam deflection with BAGMS. All anomalously deflected angles are the same when the incident wavelength is changed. **b** Phase distribution for a BAGMS at different incident wavelengths. The Δ*φ*(*x*, *λ*) represents the phase difference between *λ*_min_ and *λ*_max_ at certain surface position. **c** Simulated intensity of RCP-to-LCP scattering light vs. angle of reflection at various incident wavelengths. The angle of reflection keeps at around 22.26˚ when the incident wavelength is changed from 1200 to 1650 nm. **d** SEM image of fabricated BAGMS. **e** (*Left*) Experimentally captured CCD images of RCP-to-LCP scattering light from the BAGMS. The intensities are normalized by the maximum value in each image. (*Right*) Cross section of normalized scattering light intensity along the central line of each corresponding CCD image for RCP-to-LCP (*navy curves*) and RCP-to-RCP (*olive curves*) components

Generally, our design principle can be used to realize various flat optical components without chromatic effect like the broadband achromatic gradient metasurface (BAGMS). The design of a BAGMS to deflect a normally incident light by the same angle in a broad wavelength range is demonstrated (Fig. 5a). The building blocks are the same as those used in the above achromatic metalens, and the orientation of each unit element is designed to obey the required phase distribution:

5 $$\varphi \left( {x,\lambda } \right){\rm{ = }} - \frac{{2\pi }}{\lambda }x\sin {\theta _{\rm{d}}}$$

where *x* is the spatial coordinate and *θ*_d_ is the reflected angle of anomalous beam, namely the LCP light beam reflection with RCP incidence. To realize the phase requirement for deflecting light with the same *θ*_d_ at different incident wavelengths, the specially designed unit elements with proper orientation are implemented to compensate the phase difference Δ*φ* (*x*, *λ*), as shown in Fig. 5b. The detailed information of design is shown in Supplementary Note 5. As predicted, all the LCP light reflected angles are kept almost unchanged when the incident wavelength is changed from 1200 to 1650 nm, showing a broadband achromatic performance in anomalous beam deflection (see Fig. 5c). It is further experimentally verified by capturing the scattering light through a slight focus (see Supplementary Fig. 8 for the optical measurement). Figure 5d presents the SEM image of the fabricated sample. As shown in Fig. 5e, the beam spot keeps in the center of CCD while the incident wavelength changed, presenting a good agreement with the numerical simulation. The anomalous reflected LCP beam can only be observed with cross-polarized component, which is also verified by changing the polarization state from detection.

## Discussion

Compared with the flat optical devices using the strong resonance based unit elements, our design employs the integrated-resonant unit elements that enable the achromatic ability in a continuously broad wavelength band. Theoretically, the BAML with very high NA can also be realized with our design principle. For example, a BAML with NA as high as 0.86 is achieved with a smaller dimension (see Supplementary Fig. 9). Although the feature size of achromatic metalenses with a fixed NA is limited by the largest phase compensation, namely the largest additional phase shift *χ*, it can be further increased through structural optimization by introducing more resonances/resonators into the integrated-resonant unit elements (Supplementary Note 8). We also would like to emphasize that the proposed design principle is not only valid to the reflective metasurface devices but can also be employed for metasurface devices in a transmission scheme if one can successfully introduce transparent integrated-resonant unit elements into the devices. One of the promising candidates is high-index dielectric metasurfaces, where multiple Fabry-Pérot resonances are realized because they can be regarded as truncated cross-sectional waveguides^44^. The versatility in the selective change of phase distribution and control of chromatic dispersion is beneficial for the development of specific functionalities like opposite phase dispersion and phase mismatch in nonlinear optical cavity that are difficult to be accomplished from bulky chromatic systems.

In summary, achromatic metasurface devices which can suppress the chromatic aberration in a wide wavelength range with continuous bandwidth are demonstrated. Through exploiting geometric phase combining with phase compensation from specially designed integrated-resonant unit elements, the metasurfaces can provide the exact phase profile as the ideal requirement for broadband achromatic flat optical devices like converging metalens and metasurface deflector. Although demonstrations in the near-IR regime, the working wavelength of proposed achromatic metasurface devices can be further pushed into the visible light by using aluminum or all-dielectric based unit elements as the building blocks. Our design paves an innovate class to control the phase dispersion at will and it will greatly advance the development of innovative metadevices in full color control to reach real practical applications.

## Methods

### Sample Fabrication

A 150-nm-thick Au mirror and a 3-nm-thick Cr were first deposited on a Si substrate by e-gun evaporator. A 60-nm-thick SiO_2_ dielectric spacer is then deposited by using plasma-enhanced chemical vapor deposition (PECVD). A 100-nm-thick ZEP520A layer is consequently spin coated at 5000 rpm on the prepared substrate and then bake on a hot plate for 2 min at 180 °C. Subsequently, an Espacer layer is spin coated at 1500 rpm on the ZEP520A layer. Espacer is an organic polymer with high conductivity to reduce the positional error during the e-beam exposure process. An e-beam writing system at the acceleration voltage of 100 keV with 100 pico-ampere of current is used. The structural profile of metasurfaces is defined by the e-beam exposure and development process. The final sample will be obtained after the 3-nm-thick Cr and 30-nm-thick Au deposition and lift-off process.

### Numerical simulation

All numerical simulations were performed by using the commercial software Computer Simulation Technology (CST) Microwave Studio. For the design of unit elements, a unit cell boundary condition is employed for the simulation of reflection spectra and phase profile in an array configuration. For simplicity, perfect matched layer (PML) and periodic boundary conditions are respectively used for *x* and *y* direction for the simulation of metalenses; that is, cylindrical lenses are simulated to simply evaluate the focal length of each metalens. In the case of gradient metasurfaces, PML is employed as the boundary condition for all boundaries to mimic a free space for far-field scattering simulation. The refractive index of SiO_2_ is obtained from ref. ^45^. The permittivity of gold in the near-IR regime is described by the Lorentz–Drude model with three times damping constant larger than the bulky material.

### Data availability

The data that support the findings of this study are available from the authors on reasonable request, see author contributions for specific data sets.

## Electronic supplementary material

Supplementary Information
