# Supplementary material for: Broadband achromatic optical metasurface devices
Source: Nat Commun. 2017 Aug 4;8:187. doi: 10.1038/s41467-017-00166-7 (PMC5543157; doi:10.1038/s41467-017-00166-7)
Supplement: Supplementary file 1 — Supplementary Information [file 41467_2017_166_MOESM1_ESM.pdf]

File name: Supplementary Information
Description: Supplementary figures, supplementary tables and supplementary notes.

Supplementary Note 1

Strong coupling in MNR assemblies

Three resonant plasmonic modes are performed by peaks and dip in the efficiency spectrum once the surface plasmon modes are excited. As shown in Supplementary Fig. 1, for the resonant peaks, they are attributed to the dipole mode of each MNR. The resonant dip in efficiency spectrum associates with an abrupt change of phase modulation, which is resulting from the Fano-like resonant mode existing in such asymmetric nano-rod system with strong coupling effect. To realize a broadband achromatic metasurface device, the phase modulation must exhibit a smooth tendency within the interested wavelength range. As a result, such abrupt change of phase modulation has to be avoided in the design of integrated-resonant unit elements.

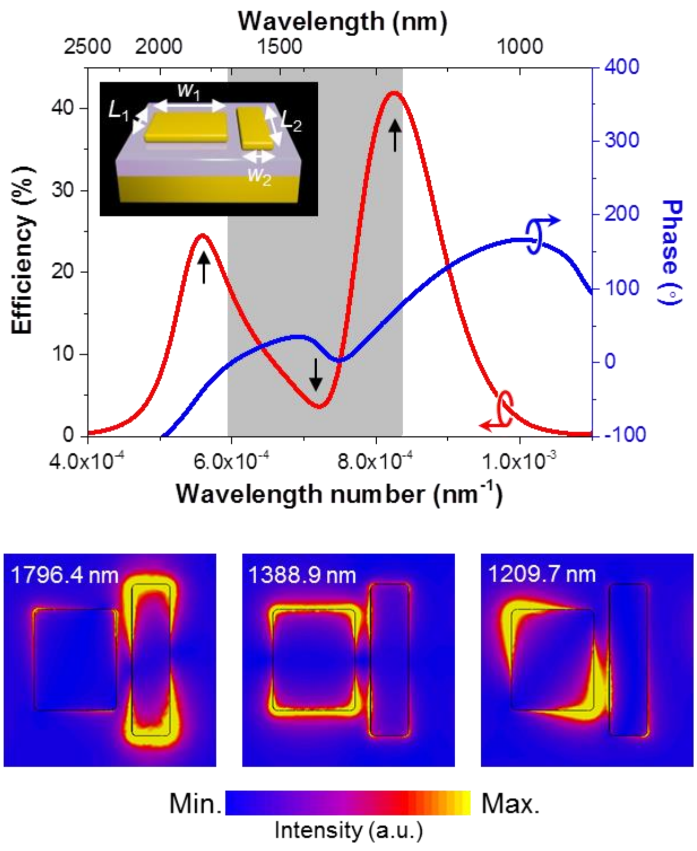

Supplementary Fig.1: Top panel shows the simulated RCP-to-LCP conversion efficiency (red curve) and phase (blue curve) spectra for strong coupled MNR assemblies. Three resonant plasmonic modes are observed from the efficiency spectrum. The black arrows represent the position of resonant modes. Bottom panel: electric field distribution of corresponding induced resonant modes. Structural dimensions: L_1 = 260 nm, W_1 = 210 nm, L_2 = 390 nm, W_2 = 100 nm.

Single MNR structure with high aspect ratio

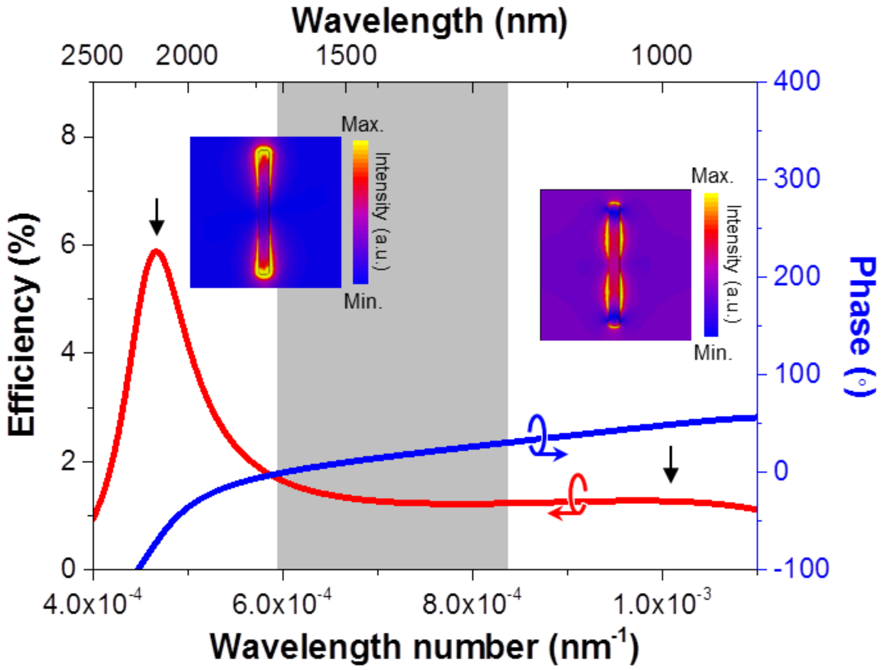

Supplementary Fig. 2: Simulated RCP-to-LCP conversion efficiency (red curve) and phase (blue curve) spectra for single MNR structure with high aspect ratio. Two resonant plasmonic modes can be observed from the efficiency spectrum, which correspond to the fundamental and high order dipole mode in the single MNR structure. The black arrows represent the position of resonant modes. Insets: electric field distribution of corresponding induced resonant modes.

Supplementary Note 2

Integrated-resonant unit elements for achromatic metasurface devices

To realize phase compensation at specific location on metasurface device interface, the phase compensation up to 360˚in a 15˚ step is achieved by using 23 unit elements. Two types of resonant features are implemented. For the case of phase compensation lower than 150˚, two resonant modes are involved, in which they are the fundamental and high order electric dipole modes along the long axis of nano-rod or the fundamental electric dipole modes along long and short axes of nano-rod. On the other hand, three resonant modes are introduced for extending the range of phase compensation larger than 150˚. Schematic of the integrated-resonant unit elements and their physical dimensions are listed in Supplementary tables 1 and 2. Supplementary Fig. 3 shows the RCP-to-LCP conversion efficiency and phase modulation of all unit elements.

Supplementary Table 1. Schematic of the integrated-resonant unit elements and their physical dimensions for phase compensation less than 150˚.

Type I	Unit element	Phase compensation (˚)	Length (nm)	Width (nm)	Number of duplicate	Gap (nm)
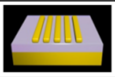	0	30	450	40	5	25
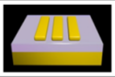	1	45	400	70	3	35
	2	60	395	130	2	40
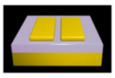	3	75	390	150	2	40
	4	90	380	170	2	40
	5	105	390	215	1	N.A.
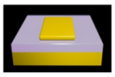	6	120	380	220	1	N.A.
	7	135	390	240	1	N.A.

Supplementary Table 2. Schematic of the integrated-resonant unit elements and their physical dimensions for phase compensation between 150˚ and 360˚.

Type II	Unit element	Phase compensation (˚)	Length 1 (nm)	Width 1 (nm)	Length 2 (nm)	Width 2 (nm)	Gap (nm)
	8	150	160	230	380	120	40
	9	165	165	235	375	120	40
	10	180	170	240	370	120	40
	11	195	180	260	380	100	40
	12	210	210	260	390	100	40
	13	225	200	260	360	100	40
	14	240	210	260	360	100	40
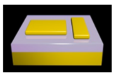	15	255	220	260	360	100	40
	16	270	225	260	360	100	40
	17	285	230	270	360	100	40
	18	300	230	270	350	100	40
	19	315	235	280	350	100	35
	20	330	240	270	345	100	35
	21	345	240	270	335	90	35
	22	360	245	270	330	90	35

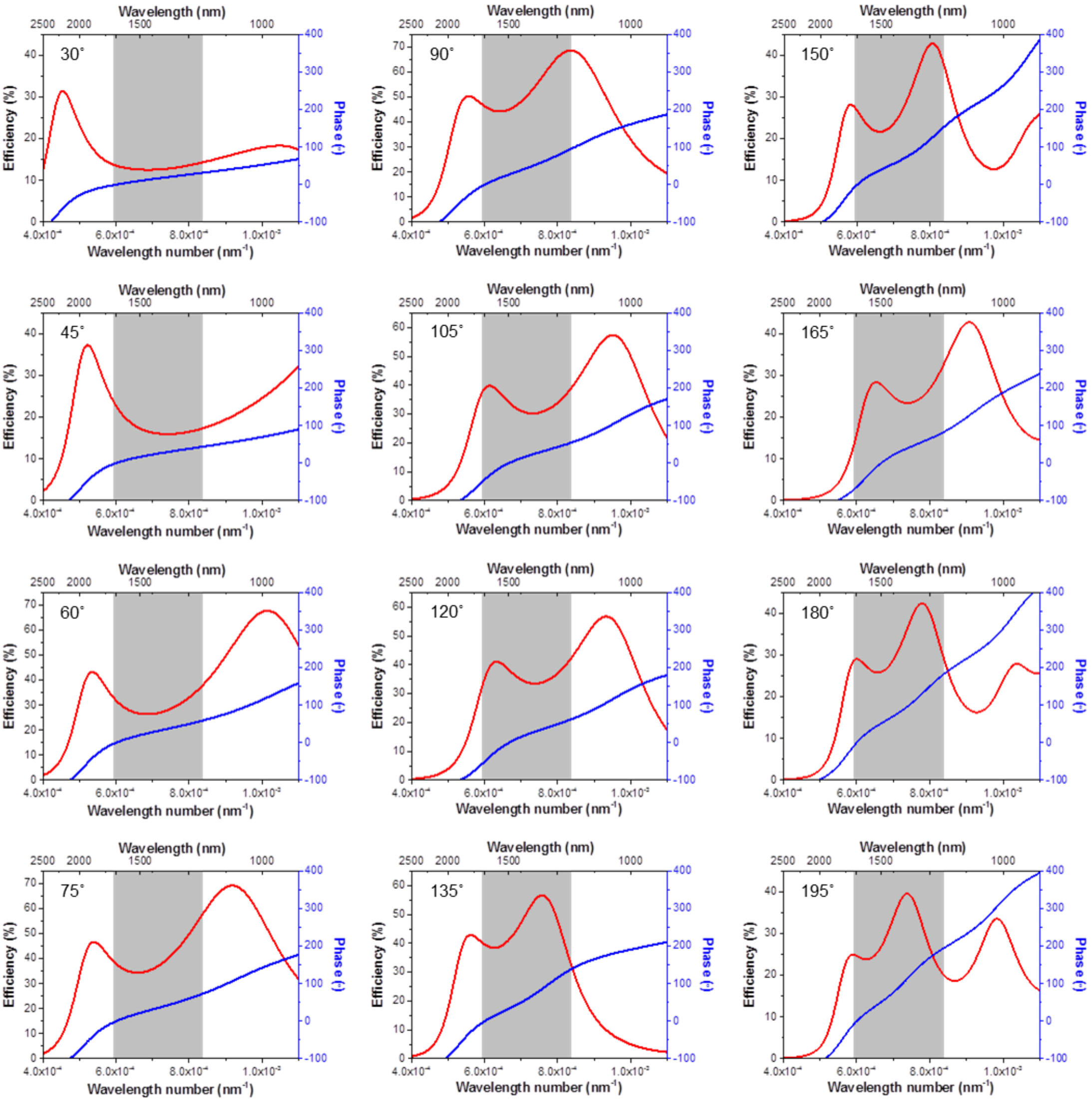
30°
90°
150°
45°
105°
165°
60°
120°
180°
75°
135°
195°
Wavelength (nm)
Wavelength number (nm^{-1})
Efficiency (%)
Phase (°)

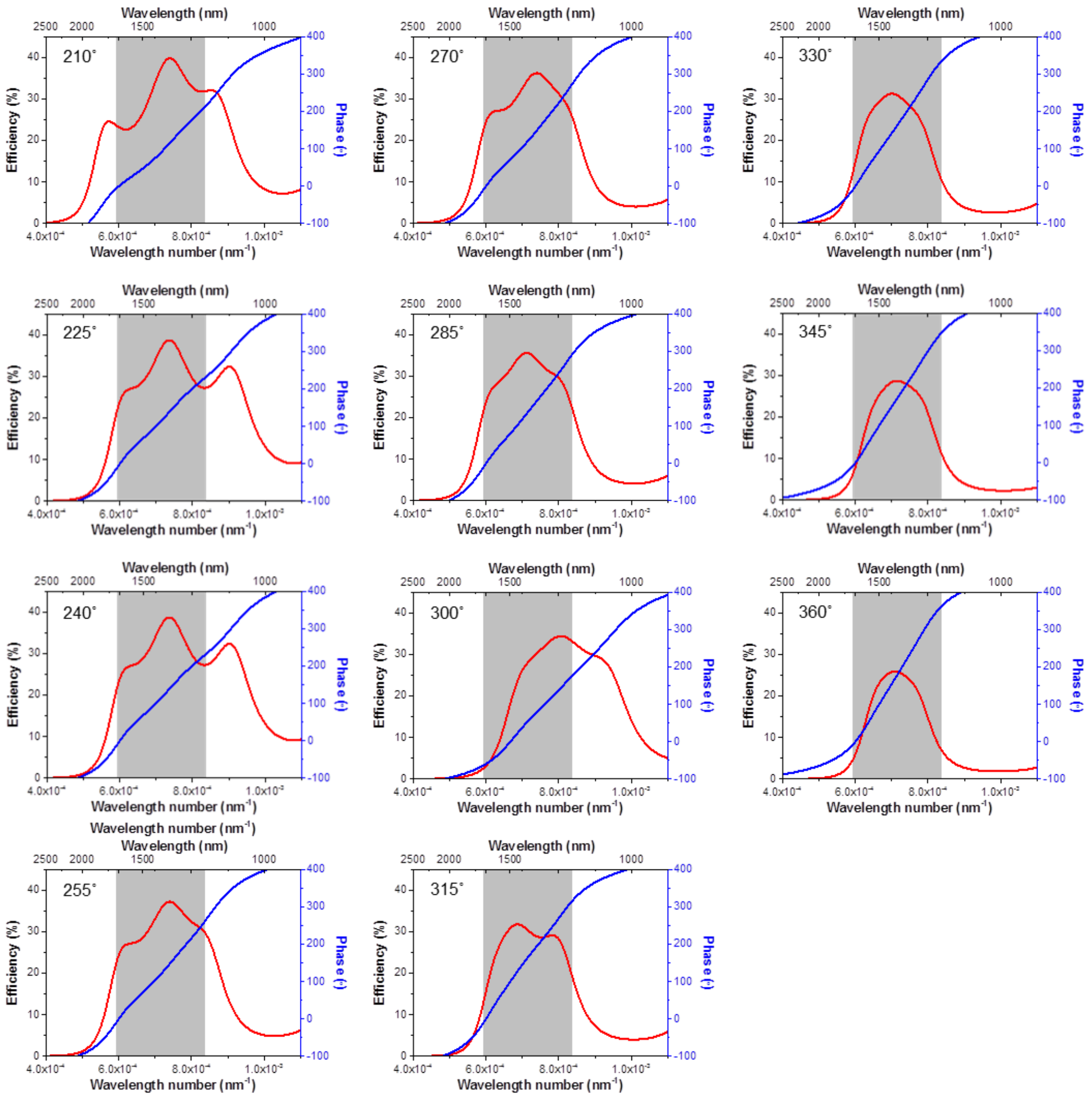

Supplementary Fig. 3: Simulated RCP-to-LCP conversion efficiency (red curves) and phase (blue curves) spectra of all 23 unit elements for phase compensation from 30˚ to 360˚, with phase interval being 15˚. The value shown in the top left corner in each spectrum indicates the phase compensation in the interested working wavelength range which is highlighted by a grey area.

Supplementary Note 3

Design parameters for broadband achromatic metalenses

The number of unit element along the radius of broadband achromatic metalens with NA = 0.324 is 41 (focal length $f = 65$ μm). During the design approach, an additional phase shift χ = 334˚ is introduced for the optimization of phase profile in this achromatic metalens. The details of physical dimensions of each unit element for this metalens is listed in Supplementary Table 3.

Supplementary Table 3. Details of each unit element along the radius of broadband achromatic metalens with NA = 0.324.

Position #	0	1	2	3	4	5	6	7	8	9
Rotating angle (˚)	326.1	325.8	325.1	323.8	322.0	319.8	317.0	313.8	303.1	298.9
Unit element	22	22	22	22	22	22	22	22	21	21

Position #	10	11	12	13	14	15	16	17	18	19
Rotating angle (˚)	294.1	288.9	288.3	282.0	275.3	259.6	251.9	243.7	237.9	228.8
Unit element	21	21	20	20	20	19	19	19	18	18

Position #	20	21	22	23	24	25	26	27	28	29
Rotating angle (˚)	219.3	209.2	198.2	187.9	176.3	146.9	134.3	126.8	122.5	105.4
Unit element	17	17	16	15	15	14	14	13	12	11

Position #	30	31	32	33	34	35	36	37	38	39
Rotating angle (˚)	91.1	74.9	53.7	43.2	27.0	7.3	351.0	332.8	315.2	296.4
Unit element	11	10	9	8	8	7	6	5	4	3

Position #	40
Rotating angle (˚)	272.2
Unit element	2

The number of unit element along the radius of broadband achromatic metalens with NA = 0.268 is 51 (focal length $f = 100$ μm). For this case, the additional phase shift χ = 335˚. The details of physical dimensions of each unit element for this metalens is listed in Supplementary Table 4.

Supplementary Table 4. Details of each unit element along the radius of broadband achromatic metalens with NA = 0.268.

Position #	0	1	2	3	4	5	6	7	8	9
Rotating angle (°)	326.1	325.9	325.4	324.6	323.4	322.0	320.2	318.0	315.6	312.8
Unit element	22	22	22	22	22	22	22	22	22	22

Position #	10	11	12	13	14	15	16	17	18	19
Rotating angle (°)	302.9	299.5	295.7	291.6	287.2	287.7	282.6	277.2	271.6	257.1
Unit element	21	21	21	21	21	20	20	20	20	19

Position #	20	21	22	23	24	25	26	27	28	29
Rotating angle (°)	250.8	244.2	240.1	232.8	225.5	217.5	209.3	200.4	191.5	183.1
Unit element	19	19	18	18	17	17	17	16	16	15

Position #	30	31	32	33	34	35	36	37	38	39
Rotating angle (°)	173.6	146.3	136.2	131.3	120.5	118.6	104.0	92.3	79.0	66.7
Unit element	15	14	14	13	13	12	11	11	10	10

Position #	40	41	42	43	44	45	46	47	48	49
Rotating angle (°)	48.2	40.6	27.3	10.8	357.8	342.9	328.5	314.2	298.7	278.1
Unit element	9	8	8	7	6	5	5	4	3	2

Position #	50
Rotating angle (°)	262.5
Unit element	2

The number of unit element along the radius of broadband achromatic metalens with NA = 0.218 is 61 (focal length $f = 150$ μm). For this case, the additional phase shift χ = 332°. The details of physical dimensions of each unit element for this metalens is listed in Supplementary Table 5.

Supplementary Table 5. Details of each unit element along the radius of broadband achromatic metalens with NA = 0.218.

Position #	0	1	2	3	4	5	6	7	8	9
Rotating angle (˚)	326.0	325.9	325.6	325.1	324.3	323.3	322.1	320.7	319.1	317.2
Unit element	22	22	22	22	22	22	22	22	22	22

Position #	10	11	12	13	14	15	16	17	18	19
Rotating angle (˚)	315.2	306.0	303.5	300.8	297.9	294.7	291.3	287.8	289.1	285.1
Unit element	22	21	21	21	21	21	21	21	20	20

Position #	20	21	22	23	24	25	26	27	28	29
Rotating angle (˚)	280.8	276.4	263.3	258.4	253.3	248.0	245.5	239.7	233.7	227.8
Unit element	20	20	19	19	19	19	18	18	18	17

Position #	30	31	32	33	34	35	36	37	38	39
Rotating angle (˚)	221.4	214.8	207.7	200.6	193.4	186.7	179.1	153.8	145.7	137.4
Unit element	17	17	16	16	16	15	15	14	14	14

Position #	40	41	42	43	44	45	46	47	48	49
Rotating angle (˚)	134.4	125.7	125.9	116.7	104.2	94.7	83.6	73.6	57.5	47.7
Unit element	13	13	12	12	11	11	10	10	9	9

Position #	50	51	52	53	54	55	56	57	58	59
Rotating angle (˚)	41.8	30.9	16.9	6.4	355.0	342.7	330.8	319.2	306.5	294.0
Unit element	8	8	7	6	6	5	5	4	3	3

Position #	60
Rotating angle (˚)	276.0
Unit element	2

Images of metalenses

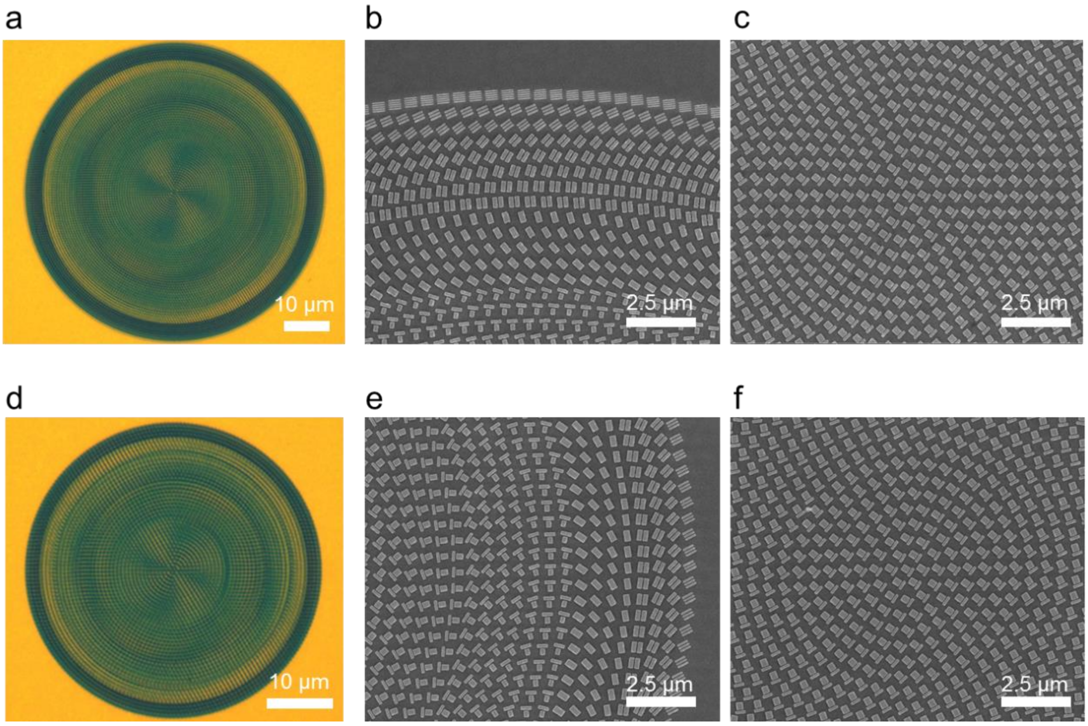

Supplementary Fig. 4: a,d Optical and **b,c,e,f** SEM images of broadband achromatic metalens with **a,b,c** NA = 0.216 and **d,e,f** NA = 0.324.

Supplementary Note 4

Design parameters for chromatic metalenses

For comparison, three geometric phase based metalenses are designed and fabricated. Supplementary Fig. 5 shows the phase profile for these three chromatic metalenses at λ = 1680 nm. In these cases the NA for each metalens at λ = 1680 nm is close to the designed BAML shown in Fig. 4a.

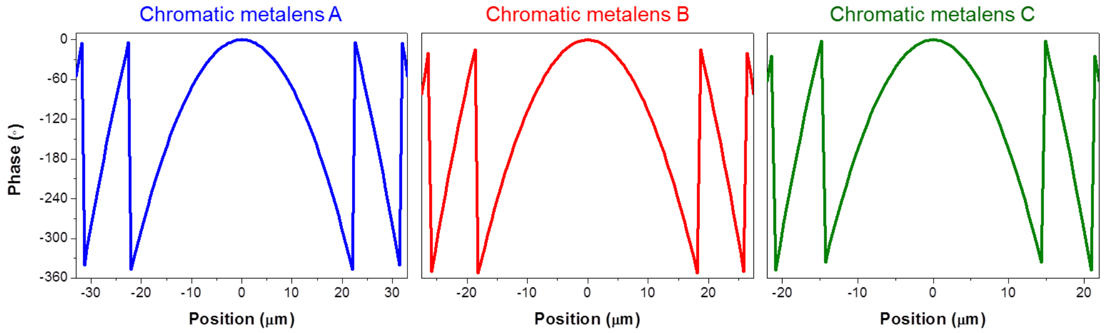

Supplementary Fig. 5: Required phase profile for three chromatic metalens at wavelength of λ = 1680 nm. The NA is designed as 0.215, 0.265 and 0.321 for chromatic metalens A, B and C, respectively.

Supplementary Fig. 6 shows the SEM images from the fabricated sample of three chromatic metalenses. The building block for all chromatic metalenses is chosen by the unit element #6 shown in Supplementary Table 1. The unit element is randomly chosen because the only issue we care here is the focal length rather than the operating efficiency. The details of physical dimensions of each unit element for chromatic metalens A, B and C are listed in Supplementary Tables 6, 7 and 8, respectively.

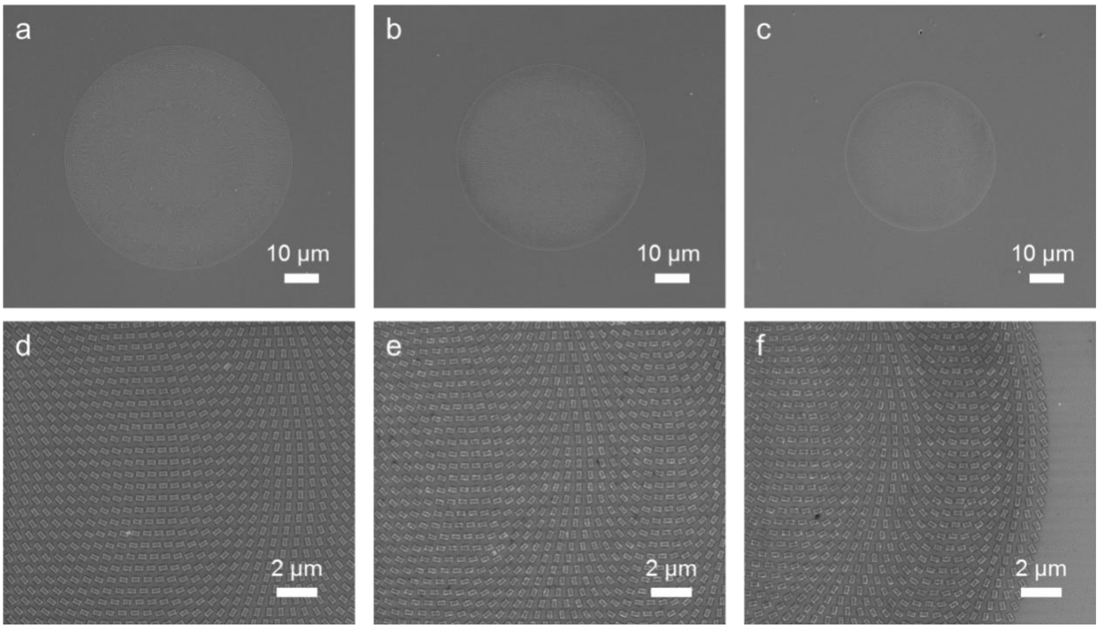

Supplementary Fig. 6: SEM images of chromatic metalens **a,d** A, **b,e** B and **e,f** C, respectively.

Supplementary Table 6. Details of each unit element along the radius of chromatic metalens A.

Position #	0	1	2	3	4	5	6	7	8	9
Rotating angle (˚)	0	0.1	0.4	1.0	1.7	2.7	3.9	5.3	7.0	8.8

Position #	10	11	12	13	14	15	16	17	18	19
Rotating angle (˚)	10.9	13.2	15.7	18.4	21.3	24.5	27.9	31.4	35.3	39.3

Position #	20	21	22	23	24	25	26	27	28	29
Rotating angle (˚)	43.5	48.0	52.6	57.5	62.6	68.0	73.5	79.2	85.2	91.3

Position #	30	31	32	33	34	35	36	37	38	39
Rotating angle (˚)	97.7	104.3	111.1	118.2	125.4	132.9	140.6	148.4	156.5	164.8

Position #	40	41	42	43	44	45	46	47	48	49
Rotating angle (˚)	173.3	2.0	11.0	20.1	29.5	39.0	48.8	58.8	69.0	79.4

Position #	50	51	52	53	54	55	56	57	58	59
Rotating angle (˚)	90.0	100.8	111.8	123.1	134.5	146.1	158.0	170.0	2.3	14.8

Position #	60
Rotating angle (˚)	27.4

Supplementary Table 7. Details of each unit element along the radius of chromatic metalens B.

Position #	0	1	2	3	4	5	6	7	8	9
Rotating angle (˚)	0	0.2	0.7	1.5	2.6	4.1	5.9	8.0	10.5	13.2

Position #	10	11	12	13	14	15	16	17	18	19
Rotating angle (˚)	16.3	19.7	23.5	27.8	32.0	36.7	41.7	47.1	52.8	58.8

Position #	20	21	22	23	24	25	26	27	28	29
Rotating angle (˚)	65.1	71.8	78.8	86.1	93.7	101.6	109.9	118.4	127.3	136.5

Position #	30	31	32	33	34	35	36	37	38	39
Rotating angle (˚)	146.0	155.9	166.0	176.4	7.2	18.3	29.7	41.4	53.4	65.7

Position #	40	41	42	43	44	45	46	47	48	49
Rotating angle (˚)	78.3	91.2	104.4	118.0	131.8	145.9	160.3	175.0	10.0	25.3

Position #	50
Rotating angle (˚)	40.9

Supplementary Table 8. Details of each unit element along the radius of chromatic metalens C.

Position #	0	1	2	3	4	5	6	7	8	9
Rotating angle (˚)	0	0.3	1.0	2.3	4.0	6.3	9.0	12.3	16.1	20.3

Position #	10	11	12	13	14	15	16	17	18	19
Rotating angle (˚)	25.1	30.3	36.1	42.3	49.1	56.3	64.0	72.3	81.0	90.1

Position #	20	21	22	23	24	25	26	27	28	29
Rotating angle (˚)	99.8	110.0	120.6	131.7	143.3	155.3	167.9	0.9	14.3	28.3

Position #	30	31	32	33	34	35	36	37	38	39
Rotating angle (˚)	42.7	57.5	72.8	88.5	104.7	121.4	138.5	156.0	174.0	12.4

Position #	40
Rotating angle (˚)	31.2

Supplementary Note 5

Design of broadband achromatic gradient metasurface

Broadband achromatic gradient metasurface exhibits a wavelength-independent angle of deflection is also realized using integrated-resonant nano-rods as the building block. The details of physical dimensions of each unit element for this broadband achromatic deflector with deflecting angle being 22.26˚ are listed in Supplementary Table 9.

Supplementary Table 9. Details of each unit element of broadband achromatic gradient metasurface.

Position #	1	2	3	4	5	6	7	8	9	10
Rotating angle (˚)	348.4	16.7	38.8	62.0	83.7	109.3	126.5	156.2	181.9	195.2
Unit element	2	4	5	6	7	8	9	11	12	13

Position #	11	12	13	14	15	16	17	18
Rotating angle (˚)	212.2	252.1	273.8	296.4	316.0	346.9	4.2	33.6
Unit element	14	15	16	18	19	20	21	22

Supplementary Note 6

Optical setup for achromatic metasurface device measurement

The focusing performances of the metalenses are characterized using a custom-built optical system, as shown in Supplementary Fig. 7.To capture the focused image at different *x*-*y* planes, several optical components including an objective (50× magnification, NA = 0.42), a lens, a quarter-wave plate and a polarization beam splitter are mounted together on a breadboard (black dashed square in Supplementary Fig. 7). First, the breadboard is properly moved to capture the image at the surface of metalens as the reference. All mounted components are then moved together along axial direction (*z*-direction) to capture the light image at different *x*-*y* planes in sequence. Finally, the light intensity profile from all captured images are stitched together to realize the intensity distribution along *z*-axis, as shown in Fig. 3b.

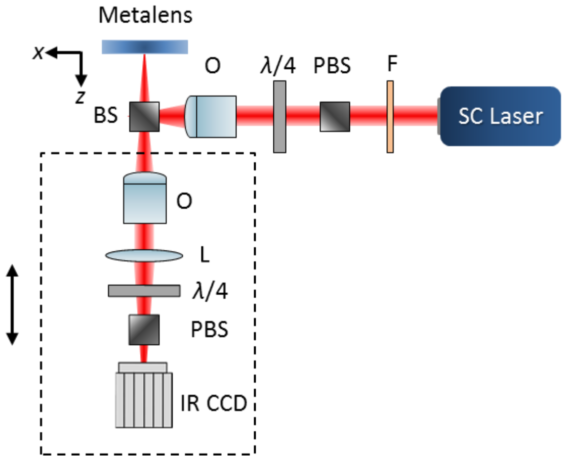

Supplementary Fig. 7: Schematic for the optical setup used for characterizing the focusing performance of the designed metalenses. The laser beam passes through a polarization beam splitter PBS and a quarter-wave plate to generate a circularly polarized light. An objective (10× magnification, NA = 0.28) is used to focus the generated circularly polarized light onto the metalens and another objective (50× magnification, NA = 0.42) is used to image the light focused by the metalens on the IR CCD (Xenics XEVA-2.5-320). An optical bandpass filter is utilized to select the incident wavelength. F: optical bandpass filter, PBS: polarization beam splitter, $\lambda/4$: quarter-wave plate, O: objective, BS: beam splitter, L: lens.

The performances of beam deflection from the gradient metasurface are characterized using another custom-built optical system, as shown in Supplementary Fig. 8. A beam splitter is

used in front of the gradient metasurface and guides the ordinary reflection to another IR CCD. To capture the anomalous beam deflection, the IR CCD is mounted along the off-axis direction with an angle θ_d from the incident light. The measured results shown in Fig. 5e can only be visualized when the angle between the incident light and the optical axis of IR CCD θ_d = 22.5˚, showing a good agreement with the theoretical predication (22.26˚). The polarization state of detected light can be directly switched between RCP and LCP by rotating the quarter-wave plate in front of the IR CCD.

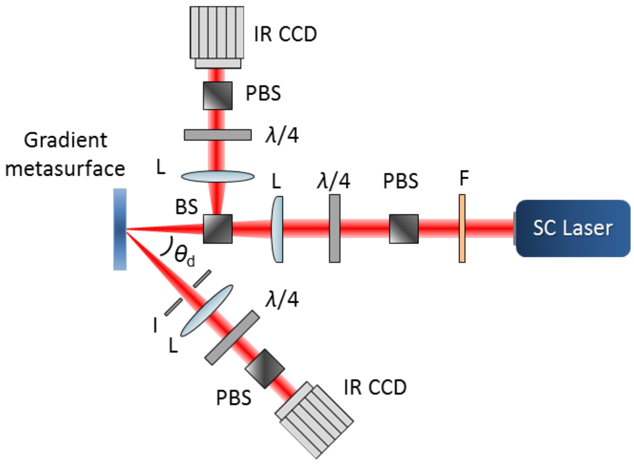

Supplementary Fig. 8: Schematic for the optical setup used for characterizing the performance of beam deflection with achromatic gradient metasurfaces. The laser beam passes through a polarization beam splitter PBS and a quarter-wave plate to generate a circularly polarized light. A lens is used to slightly focus the generated circularly polarized light onto the gradient metasurface and another lens is used to image the light deflected by the gradient metasurface on the IR CCD (Xenics XEVA-2.5-320). An optical bandpass filter is utilized to select the incident wavelength. F: optical bandpass filter, PBS: polarization beam splitter, $\lambda/4$: quarter-wave plate, BS: beam splitter, I: iris, L: lens.

Supplementary Note 7

Broadband achromatic metalens with high numerical aperture

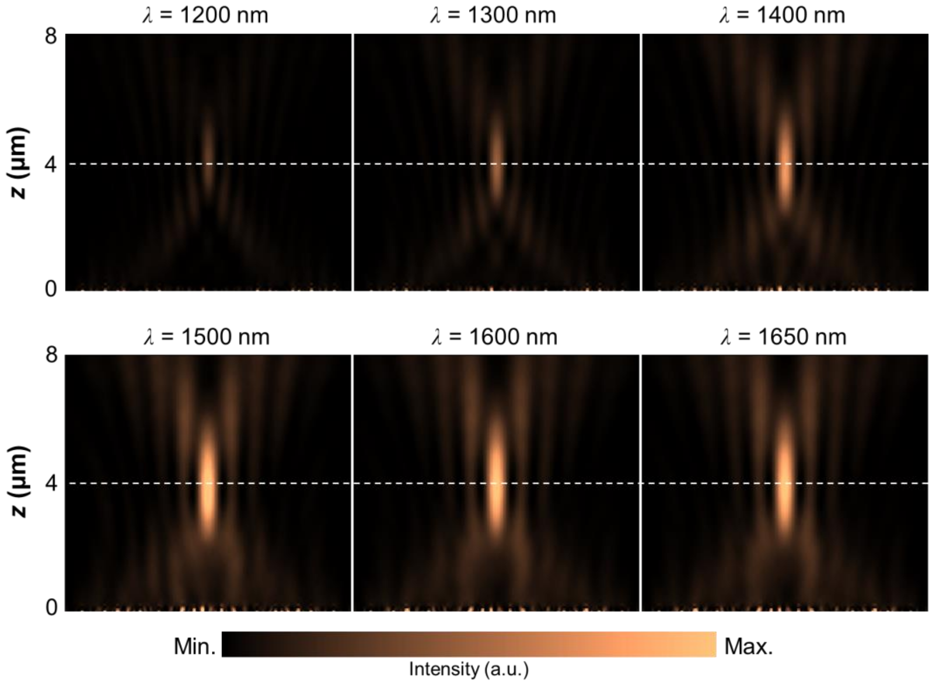

Supplementary Fig. 9: The simulated intensity profile of broadband achromatic metalens with NA = 0.86. The white dashed line denotes the position of designed focal plane.

For the proof of concept, a broadband achromatic metalens with larger numerical aperture (NA = 0.86) is designed and numerically demonstrated. As shown in Supplementary Fig. 9, the focal length is fixed at a pre-designed value when the incident wavelength is changed, showing a good performance on the elimination of chromatic effect. In this case, number of unit element along the radius of metalens is 13, while the designed focal length and phase shift is $f = 4$ μm, $\chi = 345^{\circ}$, respectively. The details of physical dimensions of each unit element are listed in Supplementary Table 10.

Supplementary Table 10. Details of each unit element along the radius of broadband achromatic metalens with NA = 0.86.

Position #	0	1	2	3	4	5	6	7	8	9
Rotating angle (˚)	333.8	329.7	310.0	283.9	263.3	226.7	190.7	134.6	106.6	57.1
Unit element	23	23	22	21	20	18	16	14	12	10

Position #	10	11	12
Rotating angle (˚)	6.2	317.5	261.9
Unit element	7	5	2

Supplementary Note 8

Design of integrated-resonant unit elements for larger phase compensation

The larger phase compensation, which enables us to design a BAML with larger diameter, can be realized by further optimizing the structural configuration of integrated-resonant unit elements. To verify this concept, we numerically provide a feasible design of unit element to obtain 450˚ phase compensation in the working wavelength range from 1200 nm to 1680 nm, as shown in Supplementary Fig. 10. To avoid the degradation on efficiency, two unit elements with the same phase compensation are designed for the construction of one BAML. For example, the first design (Supplementary Fig. 10a) shows a dip in the efficiency spectrum at wavelength ~1338.7 nm while the second one (Supplementary Fig. 10b) presents a peak at the same spectral position. Therefore, the BAML is able to present the operating efficiency at an acceptable level when both of them are taken into account in the design. The unit elements with other phase compensations can directly be realized through the same thought by carefully tuning their physical parameters like linewidth, length, gap size, structural configuration *etc*.

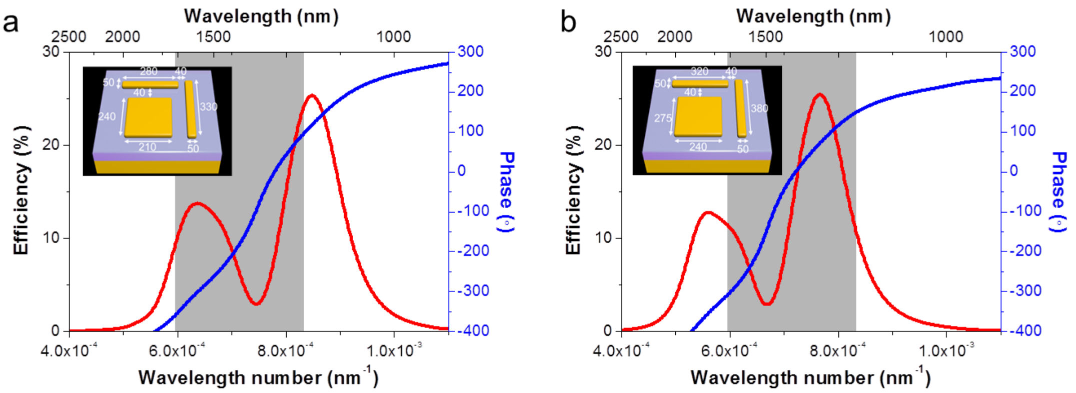

Supplementary Fig. 10: Simulated RCP-to-LCP conversion efficiency (red curves) and phase spectra (blue curves) of integrated-resonant unit elements for larger phase compensation (450˚). The grey area highlights the interested working wavelength range from 1200 nm to 1680 nm. Insets: schematics for the structural configuration and the corresponding geometric parameters in nm.
